# Enamel matrix derivative protein enhances production of matrixmetalloproteinase-2 by osteoblasts

**DOI:** 10.1186/1472-6831-14-85

**Published:** 2014-07-10

**Authors:** Seiji Goda, Hiroshi Inoue, Osamu Takeuchi, Yosuke Ujii, Eisuke Domae, Takashi Ikeo

**Affiliations:** 1 Department of Biochemistry, Osaka Dental University, Osaka, Japan; 2 Department of Physiology, Osaka Dental University, Osaka, Japan; 3 Department of Operative Dentistry, Osaka Dental University, Osaka, Japan; 4 Department of Orthodontics, Osaka Dental University, Osaka, Japan

## Abstract

**Background:**

Matrix metalloproteinases (MMPs) degrade the extracellular matrix (ECM) and regulate remodeling and regeneration of bone. Enamel matrix derivative (EMD) protein has been used clinically for periodontal regeneration, although its molecular mechanisms are not clear. We evaluated the role of matrix metalloproteinases (MMPs) in regulating EMD-dependent degradation of gelatin on oeoblast-like cell line MG63.

**Methods:**

MG-63 cells (osteoblast cell line) were incubated with 100 μg/ml EMD protein in the presence or absence of MMP-2 tissue inhibitor for 20 h followed by incubation on DQ-gelatin-coated plates for 4 h. MG-63 cells (1 × 10^6^) were preincubated with SB203580 for 30 min at 37°C and were then placed in 100 μg/ml EMD protein for 24 h. Conditioned media were collected and detected by Western blot analysis.

**Results:**

EMD protein enhanced cell-mediated degradation of gelatin, which was inhibited by the MMP inhibitor TIMP-2. Furthermore, MMP-2 was produced by MG63 cells in response to EMD protein in a P38 MAPK-dependent manner. In addition, blocking of p38 MAPK activation by SB203580 significantly inhibited generation of the active form of MMP-2.

**Conclusion:**

P38 MAPK pathway promotes expression MMP-2 in EMD activated osteoblasts, which in turn stimulates periodontal regeneration by degrading matrix proteins in periodontal connective tissue.

## Background

Two major objectives of periodontal therapy are regenerating the periodontal ligament (PDL) and rebuilding alveolar bone lost as a result of periodontal disease. Previous experimental models and clinical studies have shown that enamel matrix-derived (EMD) protein promotes generation of PDL, root cementum and alveolar bone [1-3]. EMD protein also activates osteoblasts cells in vitro, leading to a wound-healing response [4] and generation of alkaline phosphatase [5]. In addition, EMD protein regulates the production of matrix metalloproteinases (MMPs) and tissue inhibitors of MMPs (TIMPs) in gingival crevicular fluid [6,7].

Bone is continuously remodeled, and the amount of new bone depends on the balance between bone formation and resorption, which are mediated by osteoblasts, osteoclasts and osteocytes. Disturbed extracellular matrix (ECM) turnover leads to bone loss and its associated diseases, such as periodontitis. Osteoblasts are bone-remodeling cells that differentiate from mesenchymal stem cells and secrete ECM protein, which is subsequently mineralized by osteoblasts. MMPs are zinc atom-dependent endopeptidases that play a primary role in the degradation of ECM proteins [8]. Osteoblasts and osteocytes also produce MMPs such as MMP-2 and MMP-13 [7,9]. The function of MMP-2 is to degrade ECM proteins and promote remodeling and regeneration of bone tissue [10].

Mitogen-activated protein kinases (MAPKs) are important signal transducing enzymes involved in cellular regulation. Recent studies using a p38 mitogen-activated protein kinase (p38 MAPK) inhibitor showed that cytokine stimulation of MMP-2 synthesis is involved in p38 MAPK signaling [11,12].

The purpose of this study was to clarify the effects of EMD protein on the production and activation of MMP-2 using an osteoblast-like cell line, that is, MG-63. We found that EMD protein promoted the degradation of gelatin on MG-63 cells and enhanced the activation of MMP-2 in MG-63 cells. The EMD protein signaling pathways depends on p38 MAPK. These results suggest that selective regulation of MMP-2 production and subsequent activation of MMP-2 by EMD protein in MG-63 cells leads to remodeling and regeneration of periodontal connective tissue.

## Methods

### Cell line

Osteoblasts (MG-63 cell line; American Type Culture Collection, Rockville, MA) were maintained in Dulbecco’s modified Eagle’s medium (DMEM) supplemented with 10% heat-inactivated FBS (Equitech-Bio Inc., TX, USA), 2 mM glutamine and 100 units/ml penicillin/streptomycin (Invitrogen, Carlsbad, CA) at 37°C in a humidified atmosphere of 5% CO_2_ in air.

### DQ gelatin degradation assay

Coverslips were coated with 100 μg/ml quenched fluorescence substrate DQ-gelatin (Molecular Probes, Eugene, OR). MG-63 cells were incubated with 100 μg/ml EMD protein (Seikagaku-kogyo Corp., Osaka, Japan) in the presence or absence of tissue inhibitor of metalloproteinases-2 (TIMP-2; Dainippon Pharm Co., Toyama, Japan) for 20 h, followed by incubating on DQ-gelatin-coated plates for a period of 4 h. Cells were fixed with 2% paraformaldehyde in PBS. Slides were mounted with coverslips using glycerol/PBS, and examined with at 488 nm (excitation) and 533 nm (emission) using an Olympus LSM-GB200 (Olympus, Tokyo, Japan) equipped with an oil immersion lens. Differential interference contrast (DIC) was used to visualize cells cultured on the matrix.

### Western blot analysis

MG-63 (1 × 10^6^) cells were preincubated with 100 ng/ml 5 μM SB203580 (Chemicals Inc., Darmstadt, Germany) for 30 min at 37°C, and MG-63 cells were then placed in serum-free DMEM with 100 μg/ml EMD protein for 48 h. Conditioned media were collected, centrifuged to remove debris, and concentrated in Amicon Centriprep concentrators (Invitrogen) up to 10-fold. Cells were incubated in serum-free Eagle medium with 100 μg/ml EMD protein for 48 h. MG-63 cells prepared as described above were lysed with SDS-sample buffer (80 mM Tris-HCl, 3% SDS, 15% glycerol and 0.01% bromophenol blue) and sonicated briefly in order to shear DNA. Samples were separated on 10% SDS polyacrylamide gels (SDS-PAGE) under reducing conditions. Proteins were electrophoretically transferred to polyvinylidene difluoride (PVDF, Immobilon-P) membranes (Sigma-Aldrich, Inc., St. Louis, MO). Membranes were incubated for 1 h with anti-phospho-p38 antibody (Cell Signaling Technology, Danvers, MA) or anti-p38 antibody (Cell Signaling Technology) in PBS containing 0.05% Tween-20 and 10% Blockace (Dainippon Pharm Co., Toyama, Japan). Peroxidase-conjugated secondary antibody (Amersham Biosciences, Piscataway, NJ) was used at a 1:1,000 dilution and immunoreactive bands were visualized using Super Signal west pico chemiluminescent substrate (Pierce Biotechnology Inc., Rockford, IL). Signals on each membrane were analyzed by VersaDoc 5000.

#### Reverse transcription-polymerase chain reaction (RT-PCR)

Total RNA was isolated from MG-63 cells cells by RNeasy kit (QIAGEN, Valencia, CA). MG-63 cells were then placed in serum-free DMEM with 100 μg/ml EMD protein for 12 h. After denaturation of total RNA at 70°C for 10 min, cDNA was synthesized with oligo-dT primer by incubating with reverse transcriptase (Qiagen) at 50°C for 30 min. The primers for MMP-2 were 5′-TGGTTTTCCTCCATCCAGTGG-3′ (forward) and 5′-CAGGTTGTCTGAAGTCACTGC-3′ (reverse). The primers for GAPDH were 5′-ACC ACA GTC CAT GCC ATC AC-3′ (forward) and 5′-TCC ACC ACC TTG CTG CTG TA-3′ (reverse). Polymerase chain reactions were performed with Pfu polymerase (Qiagen) initiated by 1 cycle at 95°C for 15 min followed by 30 cycles at 94°C for 45 sec, 55°C for 45 sec, 72°C for 1 min and 1 cycle at 72°C for 10 min for final extension. PCR products were loaded to agarose gel, and stained with ethidium bromide. The bands were analyzed using AlphaImager™ IS-3400 software. Briefly, IDV was measured as the sum of all the pixel values after background correction in each band. The values (AVG) of each band were calculated as IND/AREA, where AREA is the size of the region that was measured. The results are shown the values of AVG_MMP1_/AVG_GAPDH_ at each time point.

## Results

### EMD protein-stimulated MG-63 cells promoted degradation of gelatin

**Figure 1:**
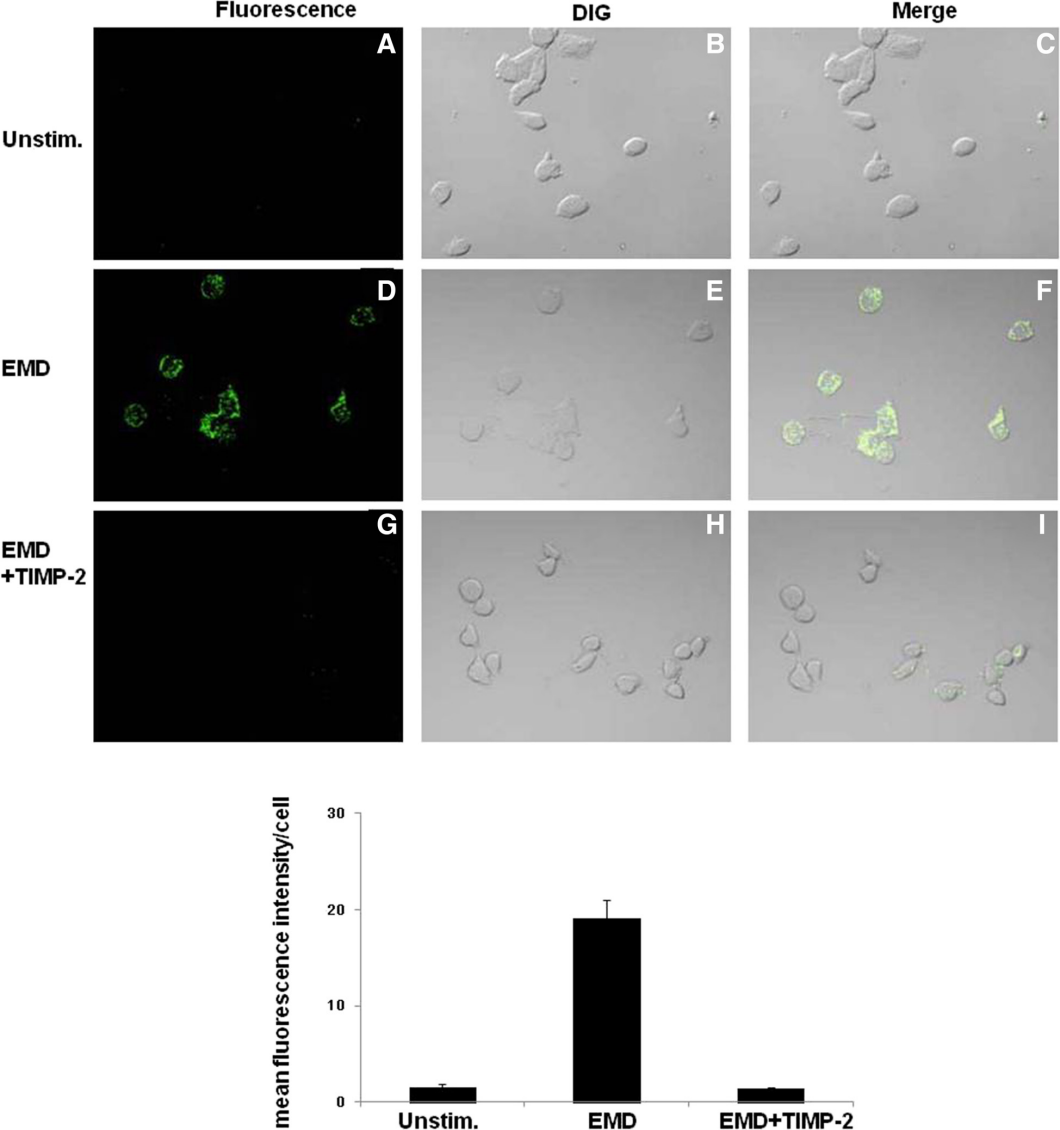
**EMD protein enhances MG-63 cells degradation of gelatin.** MG-63 cells were incubated in the presence **(D, E, F)** or absence **(A, B, C)** of 100 μg/ml EMD for 20 h, and were then incubated on glass coated with 100 μg/ml quenched fluorescent substrate DQ-gelatin for an additional 4 h. TIMP-2 (20 μM) was incubated with EMD protein and MG-63 cells as described above **(G, H, I)**. Degradation of gelatin (green fluorescence) was detected by a confocal microscope (Excitation: 488 nm; Emission: 530 nm). Images were obtained at ×40 magnification (**A** through **I**). Differential interference contrast (DIC) images are shown. Quantification of GFP in Panels **A-I** was performed densitometrically using NIH image J software. Density is depicted as mean GFP per cell.

In order to determine whether EMD protein is able to facilitate osteoblast-mediated gelatin degradation, human osteoblasts were incubated in the presence or absence of 100 μg/ml EMD protein and plated on 100 μg/ml DQ-gelatin-coated plates as described in the *Methods* (Figure 1). Degradation of DQ-gelatin was visualized in the optical section as a green fluorescent signal. Although unstimulated MG-63 cells did not produce any visible signals (panels A and C in Figure 1), EMD protein significantly enhanced degradation of gelatin (panels D and F in Figure 1). Because gelatin is a substrate for MMPs, it is possible that TIMP-2 could inhibit cell-mediated degradation of gelatin. To test this hypothesis, recombinant TIMP-2 was incubated with EMD and MG-63 cells and then cultured on DQ-gelatin. TIMP-2 almost completely eliminated degradation by EMD stimulated-MG-63 cells (panels G and I in Figure 1), indicating that degradation of gelatin is mediated by the catalytic activity of MMP, which is a key step in the promotion of periodontal connective tissue remodeling.

### EMD protein enhanced MMP-2 activity on MG-63 cells

**Figure 2:**
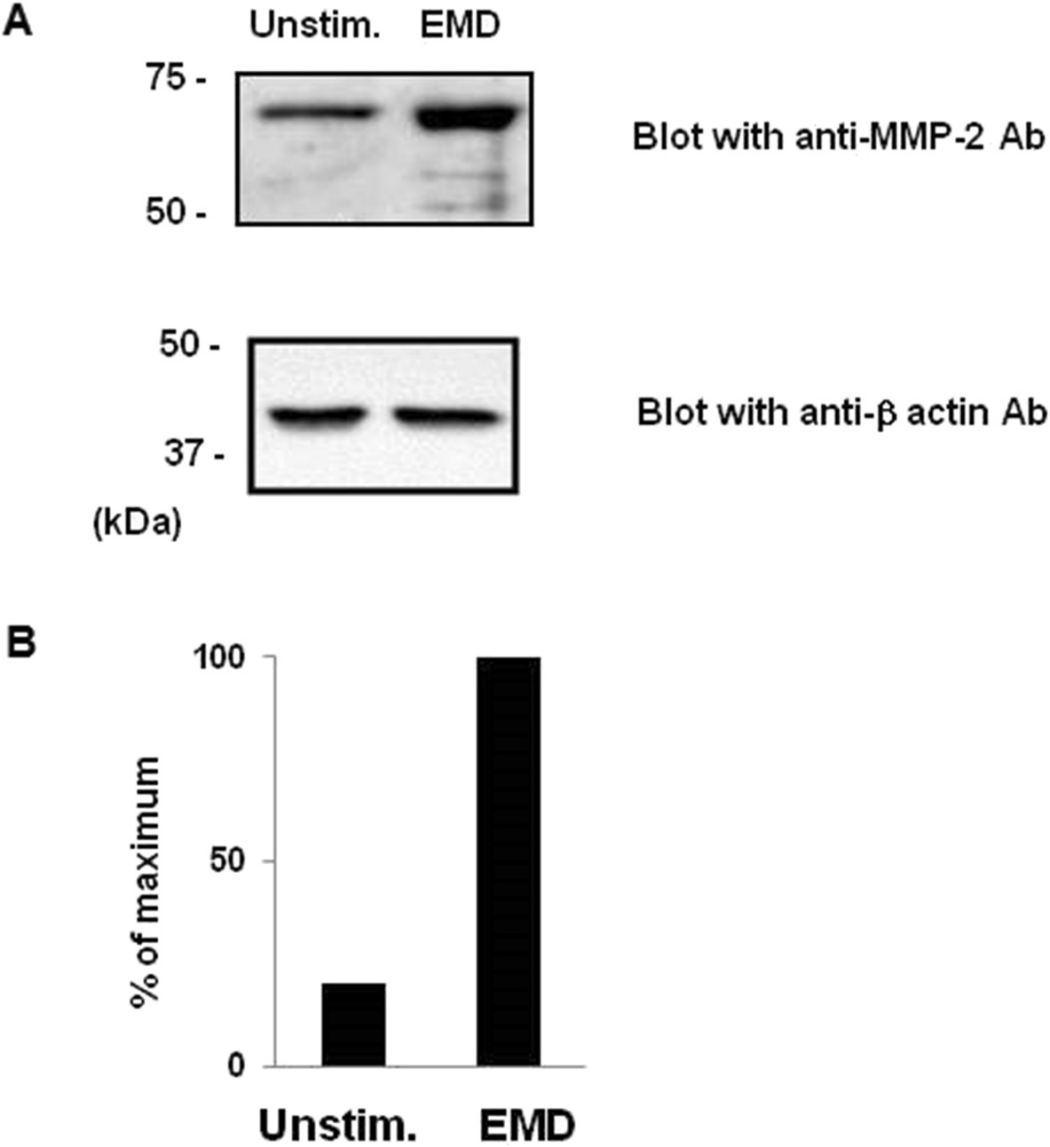
**Expression of MMP-2 from EMD protein-stimulated MG-63 cells. (A)** MG-63 cells were incubated in serum-free Eagle containing 100 μg/ml EMD protein for 24 h and conditioned media and total cell lysates were prepared as described in *Methods*. Concentrated media were separated by 8% SDS-PAGE, blotted with anti-MMP-2 antibody and visualized with Super Signal west pico chemiluminescent substrate. Molecular markers (kDa) are shown in the left column. Quantification of MMP-2 in the top panel was performed densitometrically using NIH image J software. Peak heights of each density are depicted as percent of maximum valuel. **(B)** Total RNA was isolated from MG-63 cells cultured in the presence of EMD for 24 h and subjected to RT-PCR experiments by using specific primers for MMP-2 (top) or GAPDH (bottom).

We previously showed that MG-63 cells spontaneously produced MMP-2, but not MMP-9 [10]. As shown in this study, EMD protein enhanced degradation of gelatin in MG-63 cells (Figure 1). Therefore, we also tested whether EMD protein affected the production of MMP-2 on 100 μg/ml gelatin-coated plates of MG-63 cells. EMD protein (100 μg/ml) enhanced the production of 66-kDa, 68-kDa and 46-kDa MMP-2, which correspond to the pro, intermediate and active forms of this enzyme, respectively. Importantly, when EMD protein-activated cells were cultured on gelatin-coated plates, generation of the active form of MMP-2 was also observed (Figure 2A). These results were confirmed by RT-PCR studies to demonstrate that the transcription level of MMP-2 mRNA was augmented in the presence of EMD compared to unstimulated MG-63 cells (Figure 2B).

### P38 MAPK pathway is involved in EMD protein-stimulated MMP-2 activity on MG-63 cells

**Figure 3:**
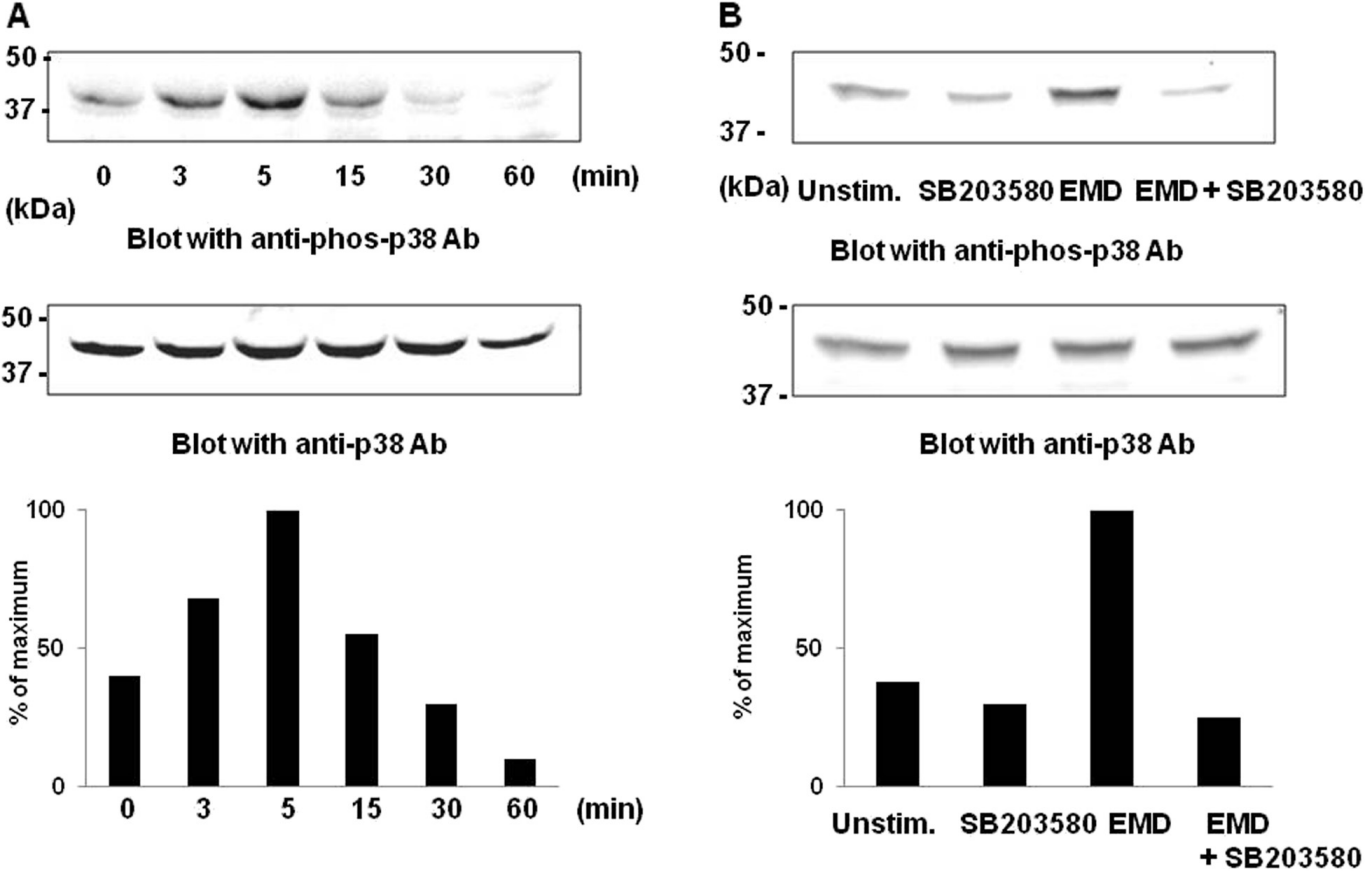
**EMD-induced activation of p38 MAPK in MG-63 cells. (A)** MG-63 cells were stimulated with 100 μg/ml EMD protein for the indicated times at 37°C. Cells were harvested, and lysates were resolved in 10% SDS-PAGE and were transferred to a PVDF membrane. Membranes were immunoblotted with anti-phospho-p38 MAPK antibody (*top*), and were then stripped and immunoblotted with anti- p38 MAPK antibody (*bottom*). Molecular markers (kDa) are shown in the left column. **(B)** MG-63 cells were treated for 30 min with SB203580 (5 μM) before stimulation with 100 μg/ml EMD protein for 5 min. Cells were harvested, and lysates were resolved in 10% SDS-PAGE and transferred to a PVDF membrane. The membrane was immunoblotted with anti-phospho-p38 MAPK antibody (*top*), and was then stripped and immunoblotted with anti-p38 MAPK antibody (*bottom*). Molecular markers (kDa) are shown in the left column. Quantification of p38 MAPK phosphorylation was performed densitometrically and corrected against the amount of total p38 MAPK protein. Peak heights of each density are depicted as percent of maximum value.

**Figure 4:**
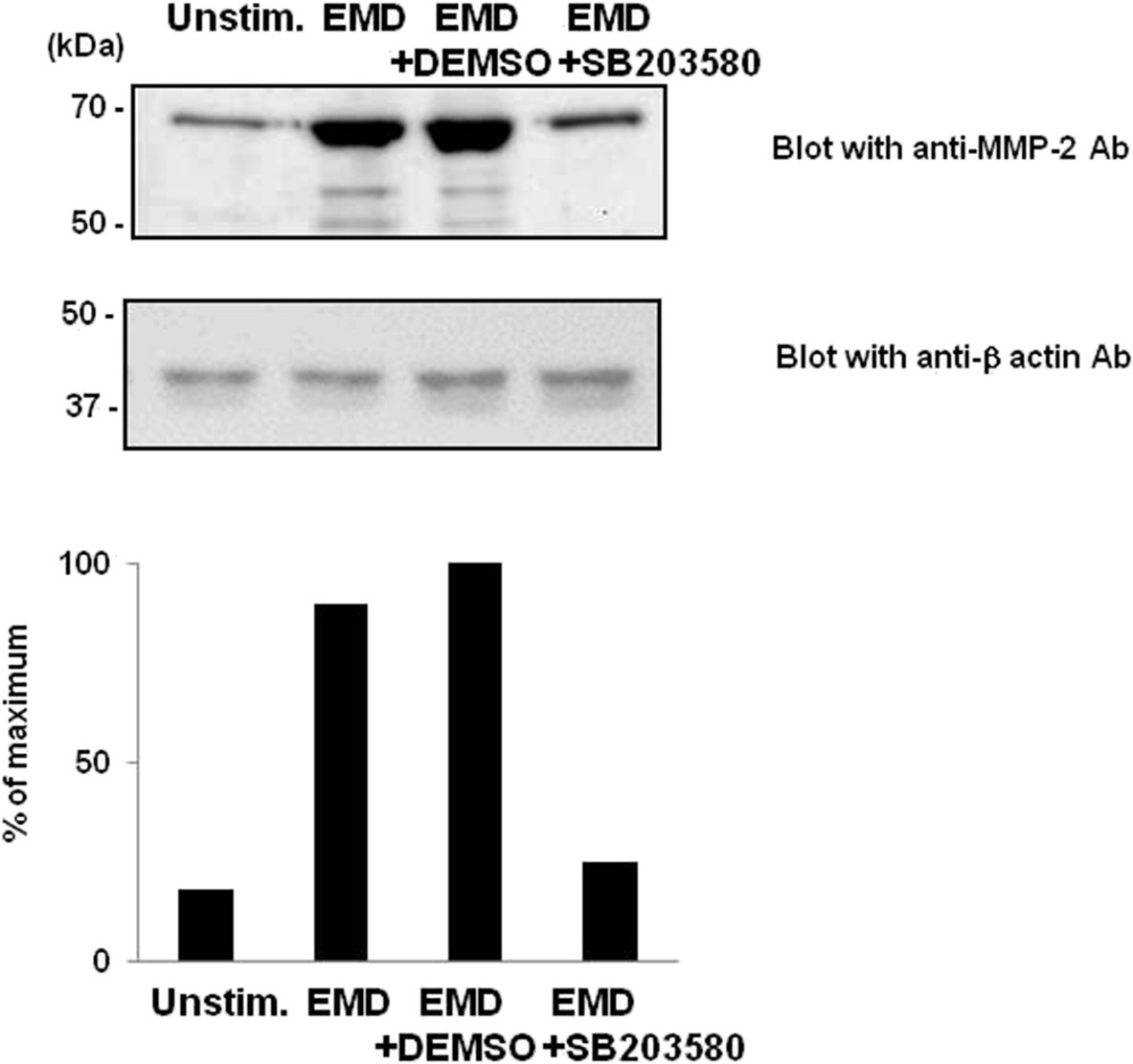
**Effects of MEK on degradation of gelatin in EMD protein stimulated MG-63 cells.** Concentrated conditioned media prepared from unstimulated MG-63 cells (*lane 1*), EMD protein-stimulated MG-63 cells (5 μM) (*lane 2*), EMD protein-stimulated MG-63 cells in the presence of DEMSO (*lane 3*), EMD protein-stimulated MG-63 cells cultured in the presence of SB203580 (5 μM) (*lane 4*) were separated on 8% SDS-PAGE. Membranes were blotted with anti-MMP-2 antibody and visualized with Super Signal west pico chemiluminescent substrate. Molecular weight markers (kDa) are shown in the left column. The same concentrated conditioned media were separated and transferred to membranes. Peak heights for each density are depicted as percent of maximum value.

Previous studies have shown that p38 MAPK regulated MMP-2 production induced by various cytokines [13]. Thus, it is possible that EMD protein also stimulates MAP kinases in osteoblasts cells to induce MMP-2 production. To test this hypothesis, we first tested whether EMD protein is able to activate p38 MAPK using Western blotting analysis. When MG63 cells were cultured in the presence of 100 μg/ml EMD protein on gelatin-coated plates, p38 MAPK activation was observed in a time-dependent manner, with the maximum phosphorylation at 5 min (top panel in Figure 3A). Total amount of p38 MAPK protein was not affected (bottom panel in Figure 3A). A synthetic specific inhibitor for p38 MAPK, SB203580, eliminated the activation of this kinase in EMD protein-stimulated MG-63 cells (Figure 3B), further indicating the activation of p38 MAPK in MG-63 cells in the presence of EMD protein.In order to further characterize the role of EMD protein-induced p38 MAPK pathway activation, MG-63 cells were pretreated with SB203580, followed by EMD protein stimulation. SB203580 significantly inhibited both production and activation of MMP-2 by EMD protein on MG-63 cells (Figure 4).

### P38 MAPK pathway is involved in EMD protein-stimulated gelatin degradation by MG-63 cells

**Figure 5:**
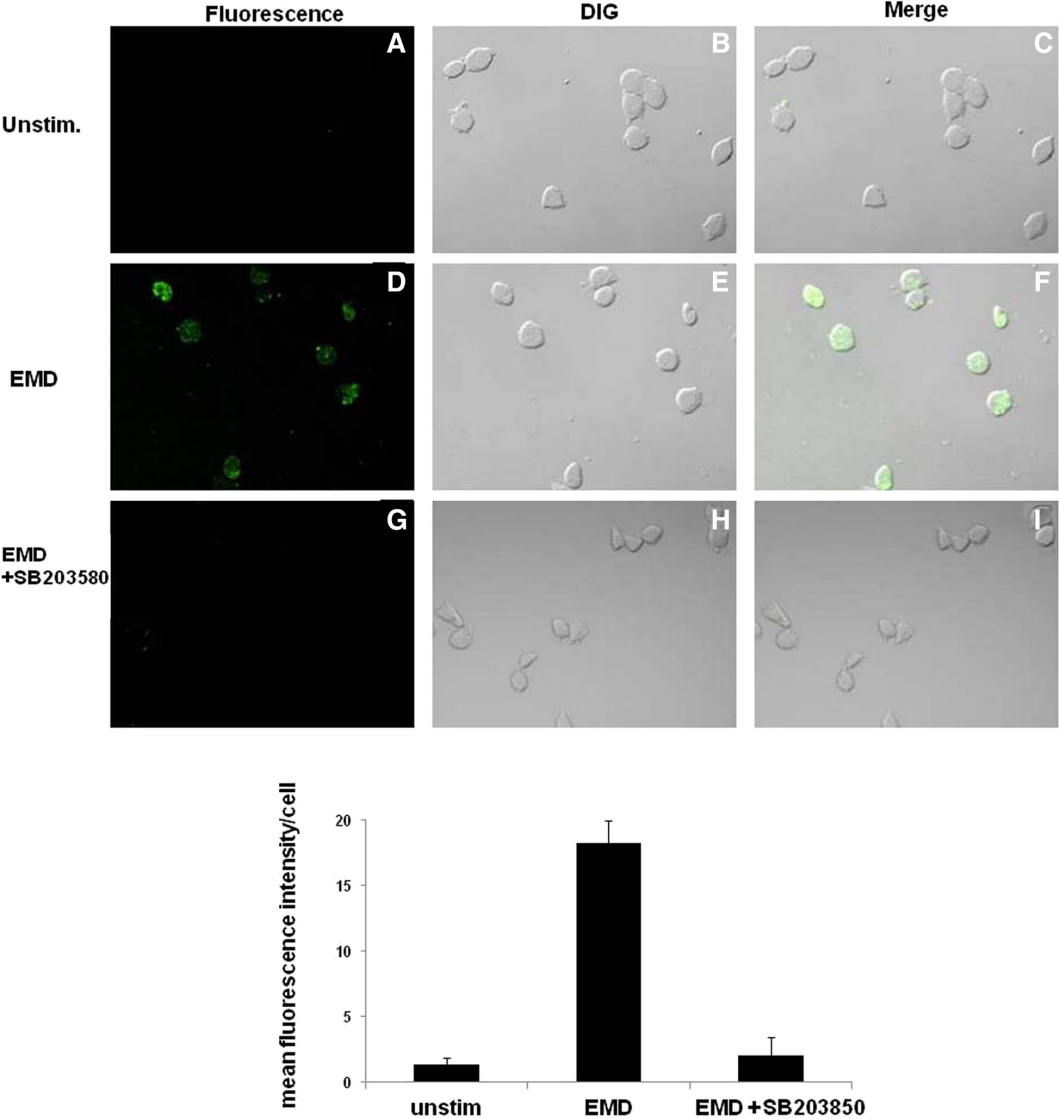
**p38 MAPK pathways are important for EMD protein-stimulated MG-63 cell-mediated degradation of gelatin.** Glass was coated with 100 μg/ml of quenched fluorescent substrate DQ-gelatin. MG-63 cells were treated for 30 min with U0126 (5 μM). Cells were incubated in the presence or absence of 100 μg/ml EMD protein for 20 h. Degradation of gelatin (green fluorescence) was detected using a confocal microscope (Excitation: 488 nm; Emission: 530 nm). Images were obtained at ×40 magnification (**A** through **I**). Differential interference contrast (DIC) images are shown. Density is depicted as mean GFP per cell.

Finally, we confirmed these results in cell-mediated gelatin degradation studies. MG-63 cells were incubated with EMD protein in the presence or absence of SB203580, and were then cultured on DQ-gelatin matrix. Consistent with results in Figure 1, EMD protein enhanced the degradation of gelatin (panels D, E, F, in Figure 5), as compared with unstimulated MG-63 cells (panels A, B, C in Figure 5). When SB203580 was co-incubated with EMD protein, degradation of gelatin was stopped, as demonstrated by a significant decrease in specific fluorescent signals (panels G, H, I in Figure 5). These findings suggest that MMP-2 is a major gelatin-degrading enzyme produced by EMD protein-stimulated osteoblasts.

These results also suggest that EMD protein stimulates matrix degradation involved in the catalytic activity of MMP-2. Furthermore, our study highlights the pivotal role for p38 MAP kinase pathways in inducing MMP-2 production from EMD protein-stimulated osteoblasts.

## Discussion

In this study, we showed that EMD protein stimulates osteoblasts to degrade gelatin in vitro and that production of MMP-2 is up-regulated in response to EMD protein stimulation. Our results also suggest that adhesion to gelatin enhances production of and activates pro-MMP-2, which is spontaneously produced by osteoblasts. EMD protein stimulates p38 MAPK signaling pathways, which in turn, induce MMP-2 production by osteoblasts. Although previous studies have shown that EMD protein applied to periodontal defects is absorbed into the denuded root dentin surface and induces periodontal tissue regeneration [14-16], the mechanisms of this action remain largely unknown. In this regard, our results show the pivotal role of MMP-2 produced from osteoblasts in response to EMD protein in facilitating periodontal connective regeneration.

MMP-2 plays a crucial role in bone remodeling and mineralization [17], and several studies have assessed the functional roles of specific MMPs [18-20]. Our results further support the importance of MMPs produced by EMD protein-stimulated osteoblasts in bone remodeling. The observations of residual degradation of ECM in the presence of TIMP-2 may be explained by the involvement of other proteases such as serine protease. Indeed, recent studies have shown that osteoblasts express surface serine protease [21]. However, the significant inhibition of gelatin degradation by TIMP-2 suggests that EMD protein enhances the production of MMPs on cell surfaces, which facilitates osteoblast-mediated gelatinolysis. In the present study, we demonstrated that MMP-2 is spontaneously produced from unstimulated osteoblasts, and that gelatinase is activated in the presence of EMD protein. In contrast, previous studies have shown that gelatinase MMP-9 was not produced by EMD protein-activated osteoblasts [10].

EMD protein contains both TGF-β- and BMP-like growth factors, which contribute to the induction of biomineralization during periodontal regeneration [22]. BMP-2 has been shown to promote bone regeneration in vivo [23], enhance alkaline phosphatase activity [24,25], and increase the production MMPs [26,27], thus suggesting that the cytokines present in EMD are necessary to promote bone regeneration. EMD protein also activates alkaline phosphatase activity [28]. It is possible that the TGF-β and BMP in EMD protein activate osteoblasts. Our data show that EMD protein enhances the production MMP-2 by osteoblasts cells and suggest that bone regeneration depends on MMP-2–activated EMD. These papers support our data [29,30]. EMD protein have decreased the levels of MMP-1 and MMP-8 in gingival crevicular fluid after flap surgery in vivo [6].

EMD-induced VEGF production is regulated by p38 MAPK in human gingival fibroblasts [31].

We also provided evidence identifying p38 MAPK as the predominant pathway for inducing MMP-2 production in EMD-stimulated osteoblasts. Previous studies have shown that this pathway is important to promote the growth of PDL cells stimulated by EMD protein [32]. Thus, the MAPK family, including the p38 MAPK, ERK and JNK pathways activated by EMD protein, plays a key role in regulating cellular functions required for periodontal regeneration [33]. EMD-induced VEGF production is regulated by p38 MAPK in human gingival fibroblasts [31]. p38 MAPK might regulate not only MMPs production, but also cytokine production in EMD stimulated periodontal ligament. Our study also indicates that inhibition of EMD protein-induced production of MMP-2 leads to significant reductions in the generation of active MMP-2, further supporting the notion that MMP-2 acts as a degradation of gelatin for MMP-2 in EMD protein-stimulated osteoblasts.

In summary, our results suggest a model in which MMP-2 plays a role in periodontal connective tissue remodeling by degrading matrix proteins and/or by activating a potent gelatinase MMP-2 in osteoblasts.

## Conclusion

EMD protein induces MMP-2 production via the p-38 MAPK-activating signalling pathways in osteoblasts, thereby stimulating degradation of the surrounding collagen, resulting in changes in ECM structure and promoting periodontal regeneration.

## Competing interests

The authors declare that they have no competing interests.

## Authors’ contributions

SG participated in planning and designing the study, in the data analysis and drafting of the manuscript. HI performed most of the laboratory work and participated in the data analysis. OS, YU, ED and TI participated in the data analysis. All authors have read and approved the final manuscript.

## Pre-publication history

The pre-publication history for this paper can be accessed here:

http://www.biomedcentral.com/1472-6831/14/85/prepub
